# Immunology of Hypertension: Pathophysiological and Therapeutic Aspects

**DOI:** 10.3390/ijms26209921

**Published:** 2025-10-12

**Authors:** Alexander Manzano, Heliana Parra, Daniela Ariza, Maria Marquina, Pablo Duran, María J. Calvo, Manuel Nava, Omar Ross, Julio César Contreras-Velásquez, Diego Rivera-Porras, Valmore Bermúdez

**Affiliations:** 1 Endocrine and Metabolic Diseases Research Center, School of Medicine, University of Zulia, Maracaibo 4001, Venezuela; amanzano_8@hotmail.com (A.M.); helianapp20@hotmail.com (H.P.); arizathings@gmail.com (D.A.); .; pabloduran1998@gmail.com (P.D.); mariacd1@gmail.com (M.J.C.); manuelnava_14@hotmail.com (M.N.); omarrosschacon@hotmail.com (O.R.); 2 Universidad de la Costa, Departamento de Productividad e Innovación, Barranquilla, Atlántico, Colombia, Barranquilla 080001, Colombia; jcontrer30@cuc.edu.co (J.C.C.-V.); drivera23@cuc.edu.co (D.R.-P.); 3 Universidad Simón Bolívar, Centro de Investigaciones en Ciencias de la Vida, Barranquilla 080001, Colombia

**Keywords:** hypertension, immune system, inflammation, cytokines, monoclonal antibodies

## Abstract

Hypertension affects over 1.39 billion people globally, causing 9.4 million deaths annually. This paper examines the intricate relationship between the immune system and hypertension, highlighting the contributions of both innate and adaptive immune responses. The innate response, involving natural killer (NK) cells, macrophages, toll-like receptors (TLRs), and dendritic cells, contributes to organ damage and inflammatory responses, exacerbating hypertension. Adaptive immunity, particularly T cells, further exacerbates vascular and renal dysfunction through the release of cytokines such as IFN-γ, IL-17A, and TNF-α, ultimately leading to multisystem damage. Therapeutic strategies targeting these immune responses are being explored, including immunosuppressants such as mycophenolate mofetil (MMF) and methotrexate (MTX), as well as monoclonal antibodies against IL-1β and TNF-α. While these strategies show promise, further research is needed to evaluate their efficacy and safety. Furthermore, this paper highlights the potential benefits of immunological approaches in managing the root causes of hypertension, offering an alternative to conventional therapies focused on the renin–angiotensin–aldosterone system. In conclusion, this work highlights the immune mechanisms in the hypertension pathogenesis, identifying them as potential therapeutic targets for enhanced management and improved patient outcomes.

## 1. Introduction

Also known as the “silent killer,” arterial hypertension (HTN) affects approximately 1.28 billion adults aged 30–79 years worldwide each year [1]. The majority of these individuals show no symptoms, making it a silent yet lethal disease that claims 9.4 million lives annually, establishing itself as one of the main risk factors for the development of cardiovascular disease (CVD). It is estimated that HTN is responsible for at least 45% of deaths associated with heart attacks and 51% of cerebrovascular accidents [2]. The increase in these figures is attributed to population growth, the aging of the population, and other risk factors such as a sedentary lifestyle, inadequate diets, obesity, and the continuous exposure to stress, among others [2].

Currently, there is no consensus on the definition of blood pressure (BP) values for diagnosing HTN, with some authors defining it as a systolic blood pressure (SBP) of 140 mmHg or higher and/or a diastolic blood pressure (DBP) of 90 mmHg or higher [3,4], while the American Heart Association suggests defining HTN as an SBP of 130 mmHg or higher and/or a DBP of 80 mmHg or higher [5]. However, it is known that the risk of developing CVD starts at values as low as 115/75 mmHg and doubles for every 20/10 mmHg increase, emphasizing the need for the prevention and proper management of HTN [4].

Despite its high prevalence and associated morbidity, only 5–10% of patients have an identified cause for their hypertension (secondary arterial hypertension). In 95% of cases, the etiology of HTN remains unknown, which is referred to as essential HTN, a clinical condition characterized by the inability to maintain a normal BP without daily treatment [6]. However, for developing effective therapeutic strategies, it is crucial to understand the pathophysiological mechanisms involved in the development of HTN, mechanisms that have not yet been fully elucidated. In this regard, HTN is generally considered a disorder involving two systems responsible for regulating the salt–water balance and cardiovascular function: the renin–angiotensin–aldosterone system (RAAS) and the sympathetic nervous system (SNS). Despite this, current treatments targeting both systems fail in about 40% of cases, suggesting that other mechanisms may be involved in the pathophysiological process of HTN [7,8].

Recently, clinical and experimental evidence has emerged supporting a contribution of immune mechanisms not only in the development but also in the maintenance of HTN and the multisystemic effects that often accompany it. This process is reflected in elevated levels of pro-inflammatory biomarkers and oxidative stress markers, while anti-inflammatory and antioxidant markers are decreased [9,10]. Additionally, one of the most prevalent comorbidities in people with autoimmune conditions is HTN [11]. However, the cause-and-effect relationship between immunity, HTN, and multisystemic effects is only beginning to be understood. It is presumed that an accumulation of immune system (IS) cells and mediators in the blood vessels, kidneys, heart, and brain promotes a chronic inflammatory response that disrupts homeostasis in these organs, leading to HTN [12,13].

This review summarizes the evidence supporting the role of the IS in the pathophysiology of the development and maintenance of HTN, advances over time, descriptions of molecular pathways, and potential therapeutic targets for the proper control and management of HTN.

## 2. Immunological Mechanisms in Hypertension: Preclinical Evidence

Over the years, epidemiological evidence has emerged establishing a link between the immune system and HTN (Table 1). However, it is still necessary today to investigate the possible pathophysiological mechanisms causing this. Given the complexity of the immune system, which comprises a wide variety of elements, there arises a need to study it by dividing it into innate and adaptive immunity, without overlooking autoimmunity and its likely role in the pathogenesis of HTN.

### 2.1. Innate Immunity

Innate immunity comprises NK cells, macrophages, receptors such as the TLRs family, dendritic cells (DCs), neutrophiles, basophiles, eosinophiles, and mast cells, among others [21,22]. All are capable of initiating an unspecific response that can become excessive and uncontrolled, damaging organs that regulate the BP, such as the brain, blood vessels, and kidneys, leading to deregulation and consequently HTN, through various mechanisms that have been described in recent research (Table 2) [23].

Starting with NK cells, which can increase their cytotoxicity under the action of interleukin 17 (IL-17), released by helper T cells (Ths), this is associated with the development of vascular dysfunction and HTN [24]. For this reason, Travis et al. infused normal pregnant rats with recombinant IL-17 and measured their mean arterial pressure (MAP) on the last day, finding a significant increase. Similarly, they observed that cytolytic NK cells and NK cells in the placenta were elevated, with a similar trend in circulating NK cells. Likewise, levels of reactive oxygen species (ROS) in the kidney increased, and acetylcholine-mediated vasorelaxation was altered, which could lead to vascular dysfunction with the deregulation of the BP [25]. These results are consistent with those of Shields et al. in 2018, who concluded that an increase in NK cells plays an important role in the pathogenesis of HTN [26].

This was corroborated by Elfarra et al., who studied three groups of pregnant rats: a control group, a second group induced with a reduced uterine perfusion pressure (RUPP), and a third group with a RUPP and NK depletion. They later measured and compared their MAP, where in normal rats the MAP had a mean of 108 ± 2 mmHg, in the RUPP rats it was 125 ± 2 mmHg, and in the RUPP rats with the NK depletion it was 122 ± 2 mmHg. Based on these results, the authors concluded that the NK depletion significantly reduces the MAP; therefore, these cells have a relevant role in the immunological mechanisms of HTN [27].

Moreover, macrophages are also very important as they are essential for the homeostasis of the cardiovascular system and the regulation of BP, as expressed by Fujiu et al. in their research. They subjected a group of mice to a pressure overload on the heart, which activated the sympathetic nervous system, innervating the kidneys and activating the epithelial cells of the renal connecting tubules through β2-adrenergic receptors. These cells, via the KLF5–S100A8–S100A9 pathway, were able to interact with tissue macrophages and induced the production of TNF-α, which in turn stimulated cytokine production within the kidney, highlighting colony-stimulating factor 2 (CSF2) [28,29].

Furthermore, angiotensin II (Ang II) also appears to play an immunological role in the pathophysiology of HTN, inducing inflammation and changes in macrophages. This effect was studied by Wu et al. in two works, one in 2018 and another in 2020, where RAW264.7 macrophages were stimulated with Ang II, finding that it caused the polarization of the macrophages to the M1 type and also promoted the release of inducible nitric oxide synthase (iNOS), TNF-α, IL-1β, and IL-6. In addition to this, there was an increase in the levels of connexin 43 (Cx43) and NF-κB (p65) in the macrophages. For this reason, they used inhibitors of these pathways and found that by inhibiting them, the effect of Ang II on macrophages was not generated, suggesting that both are involved in the inflammatory pathways induced by Ang II [30,31,32].

The activation of these macrophages is mediated by specific pattern recognition receptors that sense danger signals associated with hypertensive stimuli. Among the most relevant are the Toll-like receptors (TLRs), a well-known family of receptors that respond to molecular patterns from pathogens and injuries. While there are more than a dozen different receptors in this family, certain members have been linked to the development of HTN, particularly TLR3, which is an endosomal receptor, and TLR4, located in the plasma membrane [22,32].

For this reason, they were studied by Singh et al., where they separated three groups of mice: a wild-type group, a second group with a TLR3 deficiency (TLR3−/−), and a group with a TLR4 deficiency (TLR4−/−). They infused all three groups with either a saline solution or Ang II for three weeks, during which they measured their BP three times a week. They observed that SBP values were higher with the infusion of Ang II than with the saline, except in the TLR3−/− group. Likewise, they determined that TLR3 is the receptor linked to Ang II-induced HTN through the TRIF (toll–interleukin receptor domain-containing adapter-inducing interferon-β), while TLR4 is linked to cardiac hypertrophy but not to HTN [33].

**Figure 1:**
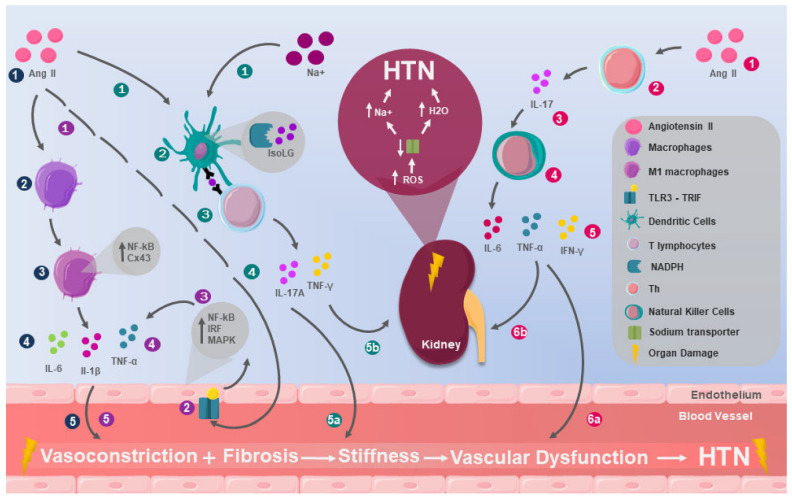
Mechanisms of innate immunity in HTN. Macrophages (dark-blue dots): 1. Angiotensin II (Ang II) appears to play an immunological role in the pathophysiology of hypertension (HTN) by 2. inducing changes in macrophages, leading to 3. macrophage polarization to the M1 type and an increase in connexin 43 (Cx43) and nuclear factor kappa B (NF–κB) levels, also promoting 4. the release of inducible nitric oxide synthase (iNOS), tumor necrosis factor-alpha (TNF-α), interleukin-1 beta (IL-1β), and interleukin-6 (IL-6), which 5. contribute to vasoconstriction and vascular fibrosis. Toll-like receptors (purple dots): 1. Ang II may also induce HTN through its effect on 2. the toll-like receptor 3 (TLR3), which is mediated by the toll–interleukin receptor domain, contains the adapter protein that induces interferon-β (TRIF). This adapter has the ability to 3. activate a signaling pathway that triggers the production of NF-κB, interferon regulatory factor (IRF), and mitogen-activated protein kinase (MAPK) and 4. culminates in the release of pro-inflammatory cytokines that enable the innate system to orchestrate an immediate local and global response, but 5. has been linked to vascular dysfunction associated with HTN. Dendritic cells (turquoise dots): The function of dendritic cells (DCs) has been affected by 1. high levels of Ang II and sodium (Na+), causing 2. the assembly of nicotinamide adenine dinucleotide phosphate oxidase (NADPH), which produces the immunogenic isolevuglandin protein (IsoLG), which is processed and 3. presented as a neoantigen to T cells, 4. triggering the production of interleukin-17A (IL-17A) and interferon-gamma (IFN-γ). In turn, these cytokines are pro-inflammatory and prohypertensive, contributing to 5a. vasoconstriction and vascular fibrosis, along with renal effects associated with a considerable increase in the number of DCs in the kidneys and elevated blood pressure, and 5b. sustained neoantigen presentation by DCs perpetuates T-cell activation, reinforcing chronic vascular inflammation and maintaining long-term hypertension. Natural killers (fuchsia dots): 1. Ang II can affect BP levels by acting on 2. T lymphocytes by stimulating the release of 3. IL-17, this interleukin increases the cytotoxicity of 4. natural killers (NKs), leading to the release of 5. IL-6, TNF-α, and interferon gamma (IFN-γ), which have effects on 6a. blood vessels generating vasoconstriction and 6b. the kidneys increasing the release of reactive oxygen species (ROS), decreasing Na+ transport; consequently, there is greater accumulation of Na+ and water (H_2_O), contributing to HTN.

In the same vein, Ao et al. studied the interrelation between signaling adapters of innate immunity receptor pathways, including TLRs and their adapter TRIF. They evaluated them in renal cell cultures from humans, pigs, cattle, goats, horses, mice, chickens, and ducks, determining that in all these species the TLRs activate a signaling pathway that triggers the production of NF-κB, IRF, and MAPK, culminating in the release of pro-inflammatory cytokines that allow the innate system to orchestrate an immediate local and global response, which has been linked with vascular dysfunction associated with HTN (Figure 1) [32,34].

While macrophages act as key inflammatory effectors and TLRs function as crucial sensors, it is the dendritic cells (DCs) that serve as the pivotal bridge between innate and adaptive immunity. As professional antigen-presenting cells, DCs have also been linked to the pathophysiology of HTN. Lu et al. proposed that the excessive stimulation of the DC leads to the greater activation of T cells in organs such as the kidney, which can cause alterations in their functioning with a subsequent deregulation of the BP. To investigate this phenomenon, they studied two groups of mice, a wild-type group and another group with a deficiency of FLT3L (Fms-like tyrosine kinase 3 ligand), an endogenous factor that stimulates the DC. They continuously infused both groups with Ang II, later determining a considerable increase in the number of DCs in the kidneys of the wild-type mice, coupled with an elevation of the MAP, compared to the FLT3L-deficient mice (FLT3L−/−) who did not show an increase in DCs or in their MAP [34,35].

Additionally, in the same study, they determined that this phenomenon causes an increase in the number of T cells and the production of pro-inflammatory cytokines in the kidney of the wild-type mice, but not in the FLT3L−/− mice. Similarly, they agreed with the results of Barbaro et al. in 2017 [36], who determined that the stimulation of the DC causes the assembly of nicotinamide adenine dinucleotide phosphate oxidase (NADPH), which produces immunogenic isolevuglandin (IsoLG), which is processed and presented as a neoantigen by the DC, promoting the differentiation of T cells and triggering the production of prohypertensive IL-17A and IFN-g [35,37].

On the other hand, Ang II is not the only one capable of triggering this process, as also described by Barbaro et al.; sodium can induce the production of IsoLG in the DC, but in this case, the process is mediated by various elements in the DC, among which the epithelial sodium channel and the sodium–hydrogen exchanger stand out. They determined that activating these channels results in the entry of calcium through the exchanger, in addition to generating the activation of protein kinase C (PKC), the phosphorylation of p47phox, and the association of p47phox with gp91phox, allowing the assembly of NADPH and the generation of IsoLG [36,38].

Finally, mast cells and neutrophils, although they have been less studied, could also be altered in patients with hypertension. It has been reported that in these patients, high levels of immunoglobulin E stimulate mast cells to release IL-6, while neutrophils produce greater amounts of superoxide anions, contributing to vascular damage and, consequently, to the pathophysiology of hypertension [39].

**Table 2:** Immunological mechanisms in hypertension: preclinical evidence.

Authors	Type of immunity	Description	Results
Elfarra et al. [27]	Innate NK Cells	Three groups of pregnant rats were studied: a control group, a group induced with RUPP, and another group induced with RUPP and NK cell depletion. NK cell levels, mean arterial pressure, fetal weight, and cytokines were monitored.	Mean arterial pressure (MAP) was measured and compared among groups: in normal rats, MAP was 108 ± 2 mmHg; in RUPP rats, 125 ± 2 mmHg; and in RUPP + NK depletion rats, 122 ± 2 mmHg.
Wu et al. [31]	Innate Macrophages	RAW264.7 macrophages were stimulated with Ang II to simulate the inflammatory process.	Macrophage polarization to the M1 type was observed, promoting the release of iNOS, TNF-α, IL-1β, and IL-6, as well as increasing the levels of connexin 43 (Cx43) and NF-κB (p65) in macrophages.
Ao et al. [34]	Innate Receptors	The interaction between innate immune receptor signaling adapters was studied in human, porcine, bovine, caprine, equine, murine, and avian renal cell cultures.	In all these species, TLRs activate a signaling pathway that leads to the production of NF-κB, IRF, and MAPK, culminating in the release of pro-inflammatory cytokines.
Lu et al. [35]	Innate	Two groups of mice were studied: a wild-type group and a group with FLT3L deficiency. Both received continuous Ang II infusions.	There was a significant increase in DC and T cells, along with pro-inflammatory cytokines in the kidneys of wild-type mice, accompanied by elevated MAP, compared to FLT3L-deficient mice (FLT3L−/−).
Rodriguez et al. [40]	Adaptive T Lymphocytes	Three groups of genetically hypertensive mice were studied: one receiving a vehicle, one infused with MMF, and a control group.	The MMF-infused group showed normalization of blood pressure; a reduction in lymphocytes, macrophages, and Ang II-positive cells infiltrating the kidney; and reduced oxidative stress.
Mikolajczyk et al. [41]	Adaptive T Lymphocytes	Ang II was chronically infused into the perivascular adipose tissue of mice.	Increased presence of T cells, particularly those with CC chemokine receptors (CCR1, CCR3, and CCR5 for RANTES), as well as increased macrophage and dendritic cell infiltration. RANTES (−/−) knockout protected against T cell infiltration.
Guzik et al. [42]	Adaptive T Lymphocytes	Ang II or DOCA was infused into RAG-1 mice, which were then transferred with T cells but not B cells.	RAG-1 mice did not develop vascular alterations upon infusion of Ang II or DOCA. However, upon T cell transfer, vascular alterations became apparent.
Sun et al. [43]	Adaptive T Lymphocytes	Hypertension was induced by angiotensin II (Ang II) infusion in mice with T cell-mediated reactivity (T cell MR) knockout.	MR deficiency in T cells reduced systolic and diastolic blood pressure, pre-existing vascular damage, and IFN-γ levels from T cells in the kidneys and aorta.
Chan et al. [44]	Adaptive B Lymphocytes	Ang II was infused into BAFFR −/− mice.	The hypertensive response to Ang II administration was attenuated.
Parra et al. [45]	Autoimmune	NOS inhibition was performed in a salt-sensitive hypertensive mouse model, followed by the infusion of HSP70 into the peritoneum.	A regulatory T cell response was observed, correcting HSP70 immune activity, reducing immune cell infiltration in renal tissue, and preventing salt-induced hypertension.

Abbreviations: IL: interleukin; tumor necrosis factor (TNF-α); interferon-γ (IFN-γ); natural killers (NKs); reduced uterine perfusion pressure (RUPP); mean arterial pressure (MAP); blood pressure (BP); angiotensin II (Ang II); inducible nitric oxide synthase (iNOS); toll-like receptor (TLR); nuclear factor kappa-light-chain-enhancer of activated B cells (NF-κB); fms-like tyrosine kinase 3 ligand (FLT3L); dendritic cells (DCs); mycophenolate mofetil (MMF); deoxycorticosterone acetate (DOCA); mineralocorticoid receptor (MR); and heat shock protein 70 (HSP70).

### 2.2. Adaptive Immunity

Adaptive or acquired immunity consists of a highly organized set of processes that eliminate or prevent threats from elements ideally foreign to the body and is capable of recognizing and remembering specific antigens, thus generating immunity. It is fundamentally made up of lymphocytes [41,46].

T lymphocytes are central orchestrators of the adaptive immune system. While their story begins with hematopoietic precursors in the bone marrow, it is within the thymus that they undergo a critical and intricate process of maturation and selection. This thymic education ensures the generation of a diverse T cell repertoire capable of recognizing foreign antigens while maintaining a tolerance to self-antigens, a fundamental balance that can be disrupted in chronic inflammatory states like hypertension. Once they complete this maturation, these cells circulate and can differentiate into various functional subtypes, including helper T cells (Th1, Th2, Th17), regulatory T cells (Treg), and cytotoxic T lymphocytes. The role of T cells in the pathophysiology of HTN varies significantly from one cell type to another, being involved in tissue dysfunction, vascular remodeling and stiffness, and renal sodium retention [47].

Over a decade ago, it was found that lymphocytes and macrophages are capable of infiltrating the kidneys of SHR, and that administering MMF caused a reduction in T and B cells, thereby attenuating the symptoms of HTN [48]. Additionally, it was demonstrated that infusions of Ang II induced an immune response mediated by Th1 cells characterized by an increase in IFN-γ and a decrease in IL-4 in the spleen and kidney [49], and thus CD4+ and CD8 lymphocytes are essential in the process of the vascular remodeling induced by angiotensin [50].

**Figure 2:**
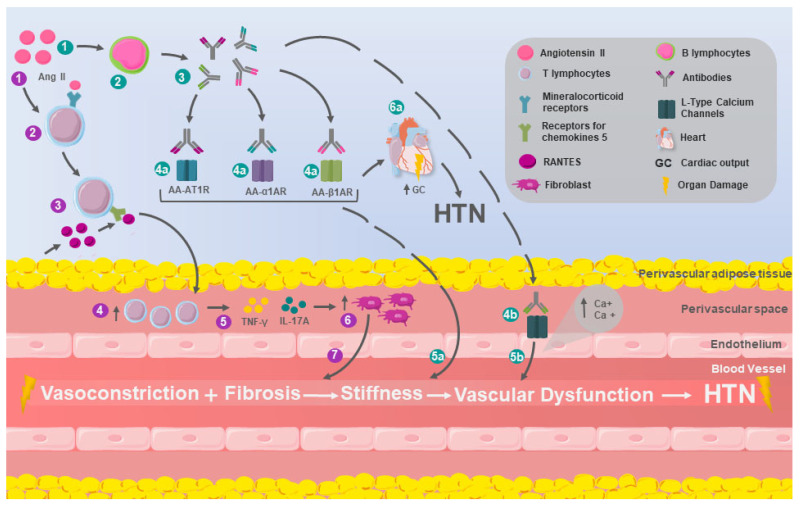
Mechanisms of adaptive immunity in HTN. T lymphocytes (purple dots): 1. Elevated levels of angiotensin II (Ang II) induce 2. an increase in the number of T lymphocytes, as well as 3. the expression of chemokine receptor-5 on them, whose ligands are the RANTES chemokines. These chemokines increase their expression in perivascular adipose tissue in the presence of Ang II. They are also capable of 4. inducing chemotaxis and the perivascular accumulation of T lymphocytes, which in turn are responsible for 5. the production of interferon-gamma (IFN-γ) and interleukin-17A (IL-17A) in the perivascular space, leading to 6. fibroblast proliferation with consequent 7. vascular fibrosis, increasing stiffness and vascular dysfunction, which are key factors in the development of hypertension (HTN). B lymphocytes (turquoise dots): 1. Ang II infusions are associated with 2. an increase in the number of B lymphocytes and the consequent 3. production of antibodies that 4a. can act as agonists of the angiotensin II type 1 receptor (AA-AT1R), the alpha-1 adrenergic receptor (AA-α1AR), and the beta-1 adrenergic receptor (AA-β1AR), whose activity has been linked to 5a. increased peripheral vasoconstriction, in addition to 6a. elevated cardiac output (CO), both of which contribute to increased blood pressure. On the other hand, 4b. other antibodies act as agonists of voltage-dependent L-type calcium channels, causing calcium accumulation in vascular smooth muscle cells, which leads to 5b. vasoconstriction, increased peripheral vascular resistance, and ultimately HTN.

Studying the perivascular leukocyte infiltration induced by Ang II and its relationship with vascular dysfunction, it was determined that chronically infusing Ang II into the perivascular adipose tissue could be observed to increase the content of T cells, especially those with receptors for chemokines CC (CCR) 1, CCR3, and CCR5 receptors for RANTES chemokines, also increasing the infiltration of macrophages and DCs. On the other hand, the elimination of RANTES was associated with protection against the endothelial dysfunction induced by Ang II, related to a decrease in vascular oxidative stress and the infiltration of IFN-γ producing T cells in the perivascular space (Figure 2) [51].

Similarly, Guzik et al. conducted research on RAG-1 mice, which lack T and B cells, observing that these mice showed a decrease in BP and did not develop vascular function alterations when infused with Ang II or deoxycorticosterone acetate (DOCA) salt. However, transferring T cells, but not B cells, restored these alterations. Furthermore, transferring T cells that lack Ang I receptors, or a functional NADPH enzyme, resulted in a decrease in BP figures in rats with Ang II-dependent HTN and a reduction in the production of ROS at the aortic level. It was also observed that Ang II significantly increased the infiltration of T cells in the perivascular adipose tissue, and that these cells exhibited a high expression of CCR5, which was associated with an increase in the intracellular adhesion molecule 1 and the expression of RANTES in the aorta. HTN was also associated with an increase in the TNF expression by T lymphocytes, thereby supporting the role of inflammation as part of the pathophysiology of HTN [42].

Moreover, studying the vascular dysfunction caused by T lymphocytes and IL-7A derived from them, Schüler et al. found that comparing mice (CD4-IL-17Aind) with control mice, elevated levels of ROS and significantly greater vascular dysfunction were observed in the first group studied, which also showed vascular fibrosis with highly proliferative fibroblasts, mainly caused by the exposure to IL-17A, finding a decrease in the pathNO/cGMP pathway, contrasting with an increase in the levels of protein tyrosine kinase 2 (PYK2) in the perivascular tissue, which is triggered by oxidative stress associated with T cells, thus demonstrating that IL-17A derived from T cells causes vascular dysfunction [52].

Another study determined that the mineralocorticoid receptor (MR) of T cells intervenes in the BP through the regulation of interferon gamma. The study was conducted on mice with a knockout of MR in T cells and HTN induced by Ang II, demonstrating that the deficiency of MRs in T cells decreases both the systolic and diastolic BP, pre-existing vascular damage, and the levels of IFNγ originating from T cells in the kidneys and aorta [43], thus showing that the inflammation produced by the activation of T cells is related to the development of HTN.

While the role of B lymphocytes in adaptive immunity is well-established, their specific contribution to hypertension development has long been a subject of considerable debate. The influential work by Guzik et al. initially appeared to resolve this question by demonstrating that while the transfer of T lymphocytes to Rag1−/− mice restored angiotensin II (Ang II)-induced hypertension, the transfer of B lymphocytes did not. This finding relegated B cells to a seemingly secondary role in the hypertension pathogenesis [42]. However, subsequent research has directly challenged this conclusion, reigniting controversy in the field. Studies using a selective B cell depletion (via anti-CD20 antibodies) or a genetic deficiency of mature B cells (BAFF-R−/− mice) have convincingly demonstrated that the absence of B lymphocytes attenuates Ang II-induced hypertension and associated vascular damage. These findings suggest a causal role for B cells, potentially mediated through the production of autoantibodies with agonistic activity against receptors such as AT1R or via the secretion of pro-inflammatory cytokines [44].

This discrepancy between key studies represents a critical gap in our understanding. It raises the fundamental question of whether B lymphocytes contribute to hypertension in a context-dependent manner or whether different experimental models reveal distinct facets of their biology. Elucidating the precise role of B cell subpopulations and their relevance in human hypertension remains a priority for future research.

### 2.3. Autoimmunity in Hypertension

The pathophysiology of essential HTN shares common characteristics with mechanisms observed in the development of autoimmune diseases, notably inflammation and oxidative stress. These are core components of the cycle linked to vascular dysfunction and kidney damage. The fundamental basis for its development involves the infiltration of T cells, B cells, macrophages, and NK cells into these organs, coupled with the effects of the cytokines they release, which lead to endothelial dysfunction and a loss of nitric oxide bioavailability, causing vasoconstriction [53].

**Figure 3:**
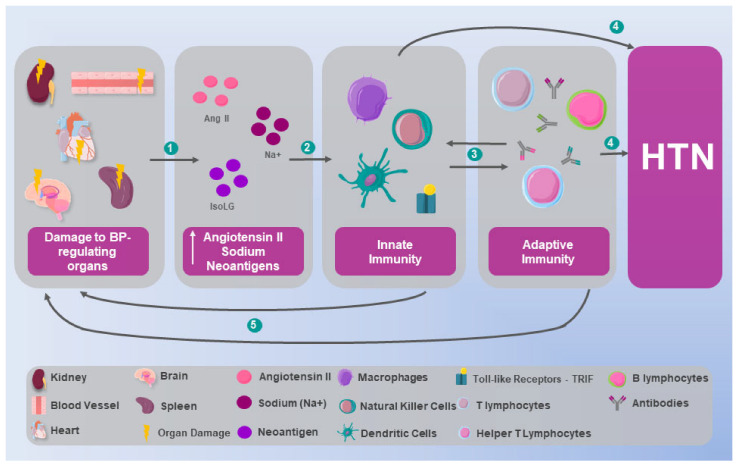
The integration of innate and adaptive immunity in HTN. When the organs responsible for blood pressure (BP) regulation are damaged, 1. the concentration of angiotensin II (Ang II), sodium (Na+), and neoantigens, such as the immunogenic isolevuglandin protein (IsoLG), increases, leading to 2. the stimulation of both the innate and adaptive immune systems, which in turn 3. interact with each other, enhancing their respective activity, 4. ultimately resulting in hypertension (HTN) through combined and individual mechanisms that include 5. damage to BP-regulating organs, perpetuating the cycle and the entire immunological mechanism of HTN.

Through experimental models in mice, Kirabo et al. [54] describe that in HTN, certain proteins oxidatively modified by γ-ketoaldehydes, known as isoketals, accumulate within DCs. This accumulation is associated with the formation by DCs of IL-6, IL-1β, and IL-23 and an increase in costimulatory proteins CD80 and CD86. Similarly, the activation of DCs promoted the proliferation of T cells, polarizing them and differentiating them into an inflammatory phenotype that involves the production of pro-inflammatory cytokines, such as IL-17, IFN-γ, and TNF-α. Clinical relevance was established through observations of elevated isoprostanes in hypertensive patients, with significantly higher levels in resistant HTN cases. These findings position isoketal-modified proteins as potential neoantigens, supported by the concomitant elevation of plasma F2-isoprostanes and mononuclear cell isoketal levels in hypertensive patients (Figure 3) [54].

The concept of oxidative stress-derived neoantigens, such as isoketal adducts, presents an elegant mechanistic framework, yet critical questions remain. It is still uncertain whether isoketals act as the primary autoimmune triggers or if they are part of a broader spectrum of autoantigens generated in the pro-oxidative environment of HTN. Furthermore, the universality of this mechanism across different hypertension subtypes (particularly salt-sensitive or treatment-resistant phenotypes) remains to be fully established. Future research should focus on characterizing a more comprehensive repertoire of hypertension-associated autoantigens, confirming their presence and pathogenic role in human cohorts, and evaluating whether antigen-specific tolerance induction could offer therapeutic benefits. Addressing these gaps is essential to validate this hypothesis and explore its potential clinical applications.

Beyond isoketal-modified proteins, other oxidative stress-related molecules have been implicated in hypertension-associated autoimmunity. Among these, heat shock proteins (HSPs) represent another important class of immunodominant molecules. It has been established that the expression of heat shock protein 70 (HSP70) is elevated in the kidneys of hypertensive mice and has been linked to the development of essential HTN, as it has properties that can induce or suppress an inflammatory response [55]. It has been hypothesized that the expression of this protein in arterial walls occurs in response to HTN, due to the biomechanical and hemodynamic stress they undergo, establishing that this phenomenon is governed by genetic variables in the individual but can be influenced by epigenetic variables [40]. A study aimed at elucidating the possible mechanisms of cell immunity involved in HTN worked with mouse models of SS hypertension by inhibiting iNOS, with a protein overload and proteinuria, and by the infusion of Ang II, which is associated with a significant increase in the expression of HSP70 in the kidney and a significant increase in the response of T cells in lymphoid tissue [45]. Another study based on mouse models of salt-sensitive HTN, obtained by inhibiting iNOS to test the relevance of immune reactivity driven by HSP70 in HTN, induced immune tolerance through the peritoneal infusion of HSP70, achieving a regulatory response of T cells, correcting the immune activity of HSP70, reducing the infiltration of immune cells in the kidney tissue, and preventing HTA caused by a high-salt diet [56].

Furthermore, it was established that there is a higher expression of genes for HSP70 in hypertensive patients compared to a control group, and this difference was also extrapolated to the expression of plasma HSP70 protein, both being statistically significant [57]. Another study, aimed at determining the interaction of HSP70 and its polymorphisms in relation to the risk presented by cooks who work with ovens of developing HTA, found that the genetic polymorphisms of the individual were capable of interacting with the environment, and that this could increase the risk of workers developing HTA in the future [58].

## 3. Inflammation and Hypertension: From Immunological Alteration to Multisystemic Involvement

**Figure 4:**
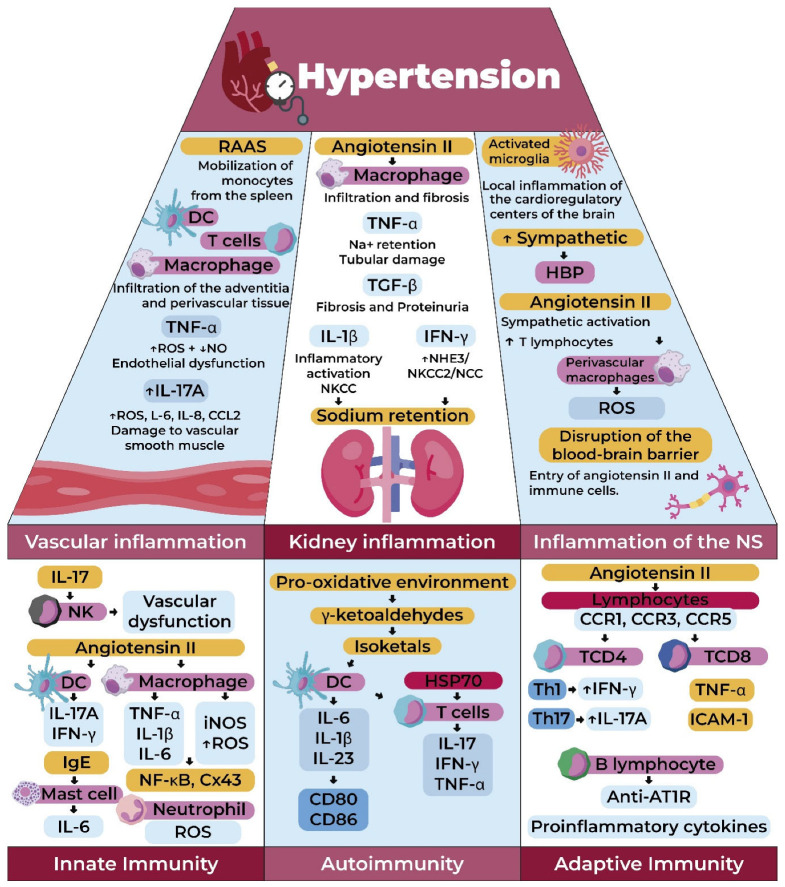
The integration of immunological mechanisms involved in hypertension. Substantial evidence indicates that the immune system plays a crucial role in the pathophysiology of arterial hypertension, acting at multiple levels and affecting various organ systems. At the core of these mechanisms lies the activation of both innate and adaptive immunity, as well as autoimmune processes. Regarding innate immunity, various cell types are involved, including natural killer (NK) cells, dendritic cells (DCs), macrophages, mast cells, and neutrophils. NK cells are activated by IL-17, which contributes to vascular dysfunction. Angiotensin II, in turn, increases the activity of DCs and macrophages, stimulating the production of large amounts of pro-inflammatory cytokines, reactive oxygen species (ROS), and inducible nitric oxide synthase (iNOS). Neutrophils also generate significant amounts of ROS, while mast cells, activated via IgE, secrete IL-6. In a pro-oxidative environment, proteins modified by γ-ketoaldehydes, known as isoketals, accumulate within DCs, promoting the release of pro-inflammatory cytokines and the upregulation of costimulatory molecules, such as CD80 and CD86, in addition to inducing T cell proliferation and activation. These T cells can also be activated by heat shock protein 70 (HSP70), which is elevated in hypertension. With respect to adaptive immunity, angiotensin II promotes the proliferation of T cells expressing chemokine receptors for RANTES (CCR1, CCR3, and CCR5), leading to the increased expression of adhesion molecules, such as ICAM-1, and the enhanced production of pro-inflammatory cytokines, including TNF-α and IFN-γ by Th1 cells and IL-17A by Th17 cells. B cells also appear to contribute by producing autoantibodies with agonist activity against receptors such as AT1R, as well as by secreting pro-inflammatory cytokines. The pro-inflammatory state described above induces inflammation in various organs, the first of which is the vascular system. The activation of the renin–angiotensin–aldosterone system (RAAS) promotes the mobilization of monocytes from the circulation and infiltration of the perivascular adventitia by immune cells such as DCs, macrophages, and T lymphocytes. This inflammatory response increases TNF-α levels, favoring ROS accumulation and reducing nitric oxide (NO) bioavailability in the endothelium, resulting in endothelial dysfunction. Additionally, increased IL-17A leads to further ROS accumulation and stimulates immune cells to produce IL-6, IL-8, and the chemokine CCL2, contributing to damage in vascular smooth muscle. Renal inflammation is another key component in hypertension. Angiotensin II activates macrophages, promoting their infiltration into renal tissue and contributing to fibrosis, while also inducing the production of several cytokines. Among these, TNF-α increases sodium retention and tubular injury; TGF-β is involved in fibrosis and proteinuria; IL-1β promotes inflammation and the activation of NK cells; and IFN-γ enhances the activity of renal sodium transporters such as NHE3, NKCC2, and NCC—all of which facilitate sodium reabsorption and thus contribute to the hypertensive pathophysiology. Nervous system (NS) inflammation has also been implicated in the development and maintenance of hypertension. Microglial activation creates a pro-inflammatory environment in brain cardioregulatory centers, stimulating a sympathetic tone and thereby raising the blood pressure. Angiotensin II not only enhances sympathetic activation but also increases the activity of T cells and perivascular macrophages, promoting ROS production both in the vasculature and within the CNS. This results in an increased permeability of the blood–brain barrier, allowing angiotensin II and immune cells to enter the CNS, thus perpetuating the vicious cycle of inflammation and sympathetic activation. Altogether, these mechanisms demonstrate how multiple components of the immune system affect various target organs, contributing to the onset, maintenance, and exacerbation of arterial hypertension, as well as the progressive damage to involved tissues. Abbreviation: RAAS: renin–angiotensin–aldosterone system; DC: dendritic cell; NK: natural killer cell; T cells: T lymphocytes; B lymphocyte: B cell; TNF-α: tumor necrosis factor alpha; TGF-β: transforming growth factor beta; IL-1β: interleukin 1 beta; IL-6: interleukin 6; IL-8: interleukin 8; IL-17: interleukin 17; IL-17A: interleukin 17A; IL-23: interleukin 23; IFN-γ: interferon gamma; ROS: reactive oxygen species; NO: nitric oxide; iNOS: inducible nitric oxide synthase; NF-κB: nuclear factor kappa B; Cx43: connexin 43; NHE3: sodium–hydrogen exchanger 3; NKCC2: sodium–potassium-chloride cotransporter type 2; NCC: sodium–chloride cotransporter; HSP70: heat shock protein 70; CD80: costimulatory molecule CD80; CD86: costimulatory molecule CD86; ICAM-1: intercellular adhesion molecule 1; AT1R: angiotensin II type 1 receptor; Th1: T helper cell type 1; Th17: T helper cell type 17; TC4: CD4+ cytotoxic T lymphocyte; TC8: CD8+ cytotoxic T lymphocyte; CCL2: C-C motif chemokine ligand 2 (also known as MCP-1); CCR1: C-C chemokine receptor type 1; CCR3: C-C chemokine receptor type 3; CCR5: C-C chemokine receptor type 5; HBP: high blood pressure; NS: nervous system; CNS: central nervous system.

Considering all the mechanisms previously mentioned, these lead to low-grade chronic inflammation, which can result in organ damage. These modifications occurring in the organs, in turn, contribute to the pathophysiology of HTN, which is why we will now provide further details (Figure 4) [59].

### 3.1. Renal Inflammation in Hypertension

The kidney plays the most critical role in BP regulation, participating in both blood volume control and vasoconstriction. Blood volume regulation occurs through the excretion of water and electrolytes—primarily sodium—while vasoconstriction occurs through the release of renin, the formation of Ang II, and the secretion of vasopressor substances. This raises the question of the potential relationship between the kidney, HTA, and the immune system. Based on evidence from biopsies performed after renal sympathectomies for the treatment of HTA [59,60] and autopsy studies of patients with severe HTA [61], inflammatory infiltration has been observed, which is why it is pertinent to describe the special changes that occur in the kidney during HTA related to the immune system.

In animal models, the case of rats exposed to an Ang II infusion for 12 days stands out. After this period, these rats showed macrophage infiltration in the renal interstitium, along with an elevated BP, interstitial fibrosis, and preglomerular fibrosis compared to the control group. However, when MMF (an immunosuppressive drug) was administered with Ang II, the previously mentioned response was prevented, suggesting a role for macrophages in salt-sensitive HTN [62]. This is supported by studies where the removal of LysM-expressing macrophages reduces the endothelial dysfunction and HTA during chronic Ang II infusion [63].

Macrophages, in turn, have the ability to release pro-inflammatory cytokines (TNF-α and IL-1β), a role also held by other immune cells. These cytokines play a fundamental role in renal damage by enhancing renal sodium reabsorption and increasing oxidative stress, among other effects [64,65]. Therefore, we will focus on describing the role of cytokines in renal damage.

Specifically, TNF-α increases the hypertensive response and associated renal injury in rodent models. Studies have shown that the specific deficit or blockade of TNF mitigates the chronic hypertensive response to Ang II [42,66] and in others where TNF was directly toxic to glomerular epithelial cells [67]. As expected, inhibiting TNF in rats attenuates the glomerular and tubular injury caused by HTN [68]. Moreover, studies involving the infusion of a TNF blocker directly into the renal interstitium have described that it can protect against salt-induced BP elevations [69]. Thus, pre-clinical studies corroborate the role of TNF in promoting sodium retention and renal damage during HTN [70].

Another important cytokine is IL-1, as mice deficient in this cytokine receptor are partially protected from HTN via the RAAS [64,65]. There are two active isoforms of interleukin 1, interleukin 1α and interleukin 1β. However, an innate signaling complex called the NLRP3 inflammasome cleaves prointerleukin 1β into its active form. These bind to the same interleukin-1 receptor (IL-1R), driving the recruitment of myeloid differentiation factor 88 (MyD88), a critical component of the TLR-dependent immune response, and several kinases that together facilitate the translocation of the p65 component of NF-κB to the nucleus where it drives the transcription of genes encoding inflammatory proteins, including TNF [71]. Components of NLRP3 have been described to exacerbate the hypertensive response to various stimuli, suggesting a role for interleukin 1 in regulating BP. Similarly to the results with TNF, the deficiency of these components mitigates the elevation of the BP and renal injury after the infusion of Ang II or mineralocorticoids [72,73]. With an additional benefit, it limits the sodium retention dependent on the Na-K-Cl cotransporter (NKCC) in the nephron, thus providing partial protection against Ang II-dependent HTN through another distinct pathway [74].

Moving beyond the previously mentioned cytokines, interferon (IFN) has also shown significant importance since the use of MMF in mice with HTN induced by DOCA and salt showed a decrease in the number of CD3+ cells in the cortex and medulla. Significant decreases in IFN-γ were also observed in the urine [75]. This cytokine, Produced by T lymphocytes and macrophages, drives and marks the differentiation of T cells into a subtype of TH1 cells and stimulates both macrophages and B cells. It also enhances sodium transport through the NHE3 transporter in the proximal tubule and through NKCC2 and NCC in the distal nephron [76].

As a result, a deficit of IFN attenuates the chronic hypertensive response to Ang II [77]. However, alterations in the heterodimeric receptor for IFN (IFNR1) did not affect the Ang II-induced elevation in BP, suggesting that signaling through the other component of the heterodimer may be sufficient to preserve IFN-dependent sodium retention. Nonetheless, signaling through IFNR1 appears to be crucial for propagating tubulointerstitial inflammation in the kidney during HTN [78].

Regarding the renal fibrosis characteristic of HTN, the transforming growth factor-β produced by renal cells and Tregs cells is key, particularly during the activation of the RAAS [79]. TGF-β triggers renal fibrogenesis by increasing the deposit of extracellular matrix proteins and inhibiting the activity of matrix metalloproteinases [80]. Consequently, the chronic administration of recombinant TGF-β1 or TGF-β2 causes renal fibrosis, proteinuria, and an elevated BP, presumably due to the loss of vascular elasticity, altered natriuresis, or both [81]. Additionally, circulating levels of TGF-β in Ang II-dependent HTN are increased [82]. On the other hand, dietary sodium, in salt-sensitive HTN, can stimulate the renal production of TGF-β [83,84]. Finally, anti-TGF-β therapy significantly reduces the BP, proteinuria, and renal fibrosis in Dahl SS rats [85].

Overall, these preclinical data indicate that the global activation of the RAAS is largely inflammatory, through the activation of inflammatory cell receptors in the kidney and other target organs. Therefore, it is proposed that the resulting target organ damage is a product of secondary immune activation, leading to the production of pro-inflammatory cytokines from infiltrating mononuclear cells, and, in turn, these cytokines mediate HTN and salt sensitivity, partly due to an impairment of renal sodium handling [64,65].

### 3.2. Vascular Inflammation in Hypertension

Vascular tissue inflammation is a hallmark of HTN, characterized by the infiltration of CD4 and CD8 T lymphocytes, macrophages, and DCs into the perivascular tissue and the adventitia of medium and large vessels, as demonstrated in experimental models [86,87,88]. In the kidney, immune cells are found around the renal arteries. The reasons for this phenomenon have not been fully elucidated, but it involves sympathetic nerve endings, and, as is well-known, perivascular inflammation is highly dependent on the CNS [37,89]. As expected, suppressing vascular inflammation has been associated with correcting HTN in various experimental models [90,91,92]. This includes the case of transferring T cells into mice genetically devoid of them, which restored the full hypertensive response to Ang II that they previously lacked [42].

Additionally, in another study, mice lacking the monocyte/macrophage chemotactic factor, the macrophage colony-stimulating factor (m-CSF), showed protection against vascular endothelial dysfunction, remodeling, and oxidative stress during Ang II-dependent HTN [63,93]. Similarly, the selective ablation of LysM+ macrophages and monocytes with the diphtheria toxin (DTX) has been shown to attenuate the hypertensive response, limiting endothelial and smooth muscle vascular dysfunction and reducing the formation of ROS in the vasculature [94].

Regarding monocytes, their migration from the spleen to the vascular subendothelium following the activation of the RAAS has been observed, thereby stimulating vascular injury [95]. Among the effectors of myeloid cells, as mentioned in this review, TNF-α is a key mediator of inflammation induced by monocytes/macrophages, playing a significant role in the hypertensive vascular pathology [96,97]. The TNF-dependent activation of nuclear factor kappa B (NF-kB) originates pro-inflammatory signaling and NADPH (NOX) that stimulates ROS generation and reduces NO production in the vasculature, resulting in the impairment of vascular endothelial function and an increased susceptibility to hypertensive stimuli [98,99,100,101]. Thus, myeloid cells derived from multiple cellular compartments participate in BP homeostasis through actions in the vasculature, producing ROS and inflammatory cytokines that contribute to vascular dysfunction and the consequent elevation of BP; i.e., the same functions of monocytes that provide innate immunity against invasive microorganisms can inappropriately elevate the BP under sterile conditions [102,103,104].

As previously highlighted regarding the role of cytokines in renal damage, other cytokines are important in vascular deterioration, such as interleukin-17A, primarily produced by CD4+ T cells [105]. This cytokine induces damage to vascular smooth muscle cells by promoting the local generation of ROS, CCL2, interleukin 8, and interleukin 6 [106]. Notably, serum levels of IL-17 are described as increasing more than threefold in hypertensive patients compared to healthy controls [107].

In mice, the systemic activation of the RAAS increases the production of IL-17 by T lymphocytes and triggers the accumulation of IL-17 protein in the medial layer of the vessel [108]. Consequently, the administration of IL-17 stimulates endothelial dysfunction and elevated BP through a ρ kinase-dependent pathway [109]. Conversely, the suppression or inhibition of IL-17A, but not blocking the alternative isoform IL-17F, inhibits renal and vascular inflammation and the elevation of BP during chronic Ang II infusion [110]. Similarly, the deletion of γ-δ T cells, a key source of IL-17A, provides protection against experimental HTN [111].

Additionally, this cytokine has renal functions not mentioned in the previous section, such as its ability to drive sodium reabsorption through the NHE3 exchanger in the proximal tubule and the sodium transporter NCC in the distal convoluted tubule [112]. Thus, the effects of IL-17A on vascular remodeling and renal sodium handling may synergistically contribute to HTN. However, the specific tissue actions and isoforms of IL-17 require further clarification, as altering this cytokine has shown detrimental effects [113].

### 3.3. CNS and SNS Inflammation in Hypertension

For a long time, renal sympathectomy has been studied as a treatment for HTN, linking the nervous system to the pathogenesis of HTA. Therefore, the role of inflammation in the CNS has been studied, and it has been found that increased inflammation in the cardioregulatory centers of the brain is associated with a greater activation of the sympathetic nervous system, resulting in an increased BP, while the inhibition of this inflammation improves HTN [114]. The sympathetic nervous system also innervates the bone marrow, spleen, and lymph nodes, releasing neurotransmitters that stimulate the mobilization and release of immune cells into the peripheral circulation, favoring, for example, the hyperactivity of T cells during HTN, which makes them more likely to increase the vascular tone, enhance the sodium transport in the nephron, and instigate autoimmune lesions in cardiovascular control tissues [115,116,117], as well as enhanced pro-inflammatory responses [118].

Regarding the effects of macrophages, in this case, it is appropriate to discuss microglia, which function as the primary innate immune cells in the CNS. This has been studied through the ablation of microglia via the intraventricular administration of DTX in mice with transgenic DTX receptor (DTR)–CD11b receptors, achieving reduced neuroinflammation and BP in multiple HTN models. This effect is supported by studies where reducing microglia lowered the expression of the glutamate receptor in the paraventricular nucleus (PVN), plasma levels of vasopressin, and concentrations of norepinephrine in the kidneys, indicating that microglia mediate the increase in the systemic BP through the modulation of neuronal excitation [119].

Ang II has been extensively studied in the CNS to understand its mechanism of action in this system. It has been observed that when centrally administered, it increases splenic sympathetic nerve activity and causes an increase in the splenic expression of pro-inflammatory cytokines, illustrating that central prohypertensive stimuli can lower the activation threshold for peripheral T cells [118]. However, T cell function is also suppressed in these models, which may help mitigate HTN. For example, ablating the circumventricular organs responsible for central sensitivity to Ang II (collectively, the AV3V) attenuates Ang II-dependent HTN as expected but also decreases the proportions of activated circulating T cells and the total number of T cells infiltrating the aorta wall [120].

The activation of the RAAS, the bioavailability of nitric oxide (NO), and subsequent sympathoexcitation play a fundamental role in HTN. In relation to this, it has been shown that there is an increased protein expression of PIN (a protein inhibitor of nNOS, neuronal nitric oxide synthase, which dissociates nNOS dimers into monomers) with concomitantly reduced levels of catalytically active nNOS dimers in the PVN of rats, mediated by Ang II. Taken together, these studies suggest that increased central levels of Ang II contribute to the enhanced expression of PIN, leading to the reduced expression of the dimeric form of nNOS, thereby diminishing the inhibitory action of NO on pre-autonomic neurons in the PVN, resulting in an increased sympathetic output [121]. This trafficking of substances and also circulating immune cells to the CNS is achieved because HTN increases the permeability of the blood–brain barrier, allowing, for example, the mentioned systemic Ang II to enter the cerebral circulation [122], and, additionally, Ang II can also activate the AT1 receptor on perivascular macrophages (PVMs) in the brain, promoting the pathogenicity of PVM actions to instigate neurovascular dysfunction through ROS production via NOX2 during chronic HTN. Thus, intrinsic CNS myeloid cells or those invading from the systemic circulation can exacerbate HTN by increasing the sympathetic output [123,124].

Finally, the sympathetic output directly reaches the kidney through the renal nerve. In this respect, bilateral renal denervation attenuates the chronic hypertensive response to Ang II, improves the hypertensive kidney injury, and limits the activation of renal T cells. The effects of the renal nerve to stimulate T cells are apparently independent of the BP since unilateral denervation allows the hypertensive response to persist and reduces T cell infiltration only in the denervated kidney [13]. Therefore, the CNS sympathetic output acts at the level of the vasculature and directly in the kidney to modulate T cell functions during HTN. In some human hypertensive patients, renal denervation similarly leads to a decrease in BP, inhibition of monocyte activation, and reduction in pro-inflammatory markers in serum. However, recent studies report that there was no evidence of the beneficial effects of renal denervation compared to standard treatments on cardiovascular morbidity and mortality [125].

### 3.4. Omics and Genetic Findings in the Immunology of Hypertension

HTN presents a complex genetic architecture that has been partially deciphered through large-scale genomic studies, particularly genome-wide association studies (GWASs). These investigations have identified over 1400 common variants across more than 900 genomic regions associated with blood pressure traits, collectively accounting for approximately 27% of its estimated heritability. Although each polymorphism contributes modestly on its own, together they reveal extensive biological networks, many of which are linked to immune and inflammatory functions. The TGF-β signaling pathway emerges as one of the most relevant in this context due to its influence on sodium re-absorption, vascular fibrosis, and the modulation of pro-inflammatory cytokines. Key genes such as SMAD, ACVR1C, and BMP2 act as mediators of these processes, while KLF14, associated with this same pathway, links immune dysregulation with metabolic dysfunctions such as insulin resistance. This functional axis between the metabolism, inflammation, and blood pressure suggests that HTN should not be viewed solely as a hemodynamic disorder but rather as an immunometabolic condition in many of its phenotypes [126,127,128,129].

The contribution of the adaptive immune system to HTN has been highlighted by variants such as rs3184504 in the SH2B3 gene, which encodes a regulatory protein in immune cells. This variant causes a missense mutation (Arg→Trp) that lowers the activation threshold of T lymphocytes, favoring a pro-inflammatory response characterized by increased IFN-γ production, the infiltration of T cells and monocytes into target tissues, and an exacerbated hypertensive response to angiotensin II. In murine models, the functional deficiency of Sh2b3 has been shown to induce a hypertensive and inflammatory phenotype dependent on hematopoietic cells. Simultaneously, integrated omics analyses have revealed the differential expression of blood pressure-related genes in tissues such as the adrenal cortex and vascular endothelium. For instance, alterations in CTNNB1 (β-catenin) are associated with autonomous aldosterone production in adrenal adenomas—a mechanism relevant in resistant hypertension that also promotes vascular inflammation. These findings suggest that certain genetic variants not only predispose individuals to physiological dysfunctions but also amplify dysregulated immune responses that perpetuate target organ damage [127,128,130].

Additionally, multiple GWAS-identified genes have been linked to vascular tone regulation and innate immunity. VEGFA, FGF9, and APOE are involved in angiogenesis, leukocyte adhesion, and endothelial integrity, while UTS2R and ARHGEF25 modulate smooth muscle contraction through Rho GTPase-dependent pathways. The influence of PRDM6, an epigenetic modulator expressed in smooth muscle and ren-in-producing cells, further strengthens the connection between vascular development and the hormonal regulation of blood pressure. These observations have led to the development of polygenic risk scores (PRSs), which integrate multiple genetic variants to predict the individual risk of HTN and associated cardiovascular events. Such scores have shown correlations with lifestyle traits such as obesity, alcohol consumption, and insulin sensitivity, highlighting a deep interaction between genetic predispositions, environmental factors, and immunoinflammatory mechanisms in the pathophysiology of hypertension [127,128,130,131].

## 4. The Management of Hypertension as an Immunological Disease: Are There Possibilities for New Therapeutic Targets?

Currently, pharmacological therapy is the primary means of controlling BP; there are multiple first-line drugs, including angiotensin receptor blockers (ARBs), known for modulating the RAAS and protecting vulnerable organs by controlling inflammation and reducing oxidative stress [132]. Studies evaluating the effect of candesartan on plasma levels of inflammatory mediators such as Soluble Intercellular Adhesion Molecule-1 (sICAM-1), interleukin-6 (IL-6), and high-sensitivity C-reactive protein (Hs-CRP) have found that levels of these mediators are significantly lower after treatment, with a significant decrease in the systolic and diastolic blood pressure [133,134,135]. Conversely, a prospective study in a general population in Switzerland, where levels of C-reactive protein (CRP), interleukins 1β (IL-1β), and 6 (IL-6), and tumor necrosis factor alpha (TNF-α) were studied and categorized, found no differences in levels of these inflammatory markers between participants who took ARBs and those who did not [136]. However, none of the participants in the ARB group were on a monotherapy, and the methods used to measure inflammatory markers were serum-based, leaving an open window for local anti-inflammatory effects in organs to differ.

Although the aforementioned drugs are well-established and approved for the clinical management of arterial hypertension, recent years have seen growing interest in exploring innovative therapeutic strategies. These novel approaches aim to address hypertension from its underlying origin as an inflammatory condition. As a result, emerging lines of investigation are focusing on immunosuppressive agents, cytokine-targeted therapies, and the modulation of the gut microbiome as potential avenues for a more comprehensive and targeted hypertension treatment.

### 4.1. Immunosuppressant Agents

#### 4.1.1. Mycophenolate Mofetil

Clinical HTN is undoubtedly associated with immune activation, a fact we have sufficiently described. Human studies evaluating the effect of immunomodulatory drugs in patients with an immune pathology plus HTA have shown encouraging results. For example, a study described that eight patients with essential HTN who received MMF for coexisting rheumatoid arthritis or psoriasis without modifying their antihypertensive treatment or diet had a decrease in BP during the MMF treatment associated with a reduction in urinary TNF-α, highlighting that the BP returned to previously high levels when MMF was discontinued [137], suggesting that the improvement in HTN was the result of immunosuppression. Similarly, other studies have reported the effect of immune therapies on HTN as a comorbidity of other classic autoimmune diseases, such as SLE, psoriasis, periodontitis, and rheumatoid arthritis [53,137], reporting favorable effects on BP. However, there is a reluctance to treat primary HTN from an immunological approach [138]. Therefore, the risk–benefit ratio of immune-targeted therapies for primary HTN must be evaluated, and, to date, this risk has been described as probably too high to justify a change in the current treatment [139].

#### 4.1.2. Methotrexate

Attempting to consider a treatment that directly involves the immune system without many adverse effects, the use of methotrexate (MTX) (a first-line antirheumatic drug) as part of antihypertensive treatment has been proposed for its discreet side effects, comparable to those of classic antihypertensives [140,141]. As reported by a study comparing patients with rheumatoid arthritis on the MTX treatment, they had a significantly lower central and peripheral BP over 24 h compared to those not taking MTX [142]. Additionally, low doses of MTX (target doses of 15 to 20 mg per week) have been studied for the prevention of atherosclerotic events in patients with a history of myocardial infarction or multivessel coronary disease who also have type 2 diabetes mellitus or metabolic syndrome. After more than two years of follow-up, it was shown that MTX did not produce lower levels of IL-1β, IL-6, CRP, or components of the metabolic syndrome than the placebo [143].

### 4.2. Selected Anti-Cytokine Therapies

#### 4.2.1. Anti-TNF-α

Anti-TNF-α therapy, among other approaches, has gained increasing attention in recent years for its potential blood pressure-lowering effects. A recent study investigating the effect of anti-rheumatic therapies on the BP in patients with recent-onset RA revealed significant findings. Over a one-year monitoring period, patients treated with infliximab (IFX) experienced a notable decrease in BP, specifically an average reduction of 4.7 mmHg in SBP and 3.9 mmHg in DBP. It is important to note that upon the discontinuation of the IFX treatment, BP levels tended to return to their initial pre-treatment values [144]. Another study, conducted by Yoshida et al., investigated the impact of IFX on BP in 16 patients diagnosed with RA. Using 24 h ambulatory BP monitoring, they observed a significant decrease in BP after two weeks of the IFX treatment. Specifically, reductions were noted in the morning BP (from 129.7 ± 19.7 to 116.9 ± 13.4 mmHg) and daytime BP (from 131.8 ± 15.1 to 122.5 ± 13.7 mmHg). Notably, only 44% of the patients in this study had hypertension, indicating that the observed BP reduction occurred in both normotensive and hypertensive individuals [145].

In contrast, a randomized controlled trial that evaluated whether combining MTX with IFX is more effective than MTX alone in preventing atherosclerosis and arterial stiffness in patients with early RA concluded that while MTX plus IFX led to a more substantial reduction in the pulse wave velocity, a key indicator of arterial stiffness, it did not find significant differences in the BP of the studied patients after six months of treatment [146]. Similar findings were reported by an Australian study that administered TNF-α inhibitors, specifically etanercept, adalimumab, or infliximab, to RA patients for six weeks. No significant changes were observed in the blood pressure values or improvements in the arterial stiffness among the patients studied [147].

#### 4.2.2. IL-1β

On the other hand, therapies targeting IL-1β have demonstrated encouraging outcomes. Notably, canakinumab has been shown to reduce key inflammatory biomarkers in patients with a history of myocardial infarction, as well as leading to a significantly lower rate of recurrent cardiovascular events than the placebo group, but it was also associated with a higher incidence of fatal infections than the placebo [148]. Recently, the CANTOS (Canakinumab Anti-Inflammatory Thrombosis Outcome Study) provided the opportunity to test whether IL-1β inhibition would reduce BP, prevent incident HTN, and modify the relationships between HTN and cardiovascular events. In this study, 10,061 patients with a previous myocardial infarction and Hs-CRP ≥ 2 mg/L were randomized to canakinumab 50 mg, 150 mg, 300 mg, or a placebo. A total of 9549 trial participants had BP records during the follow-up; of these, 80% had a pre-existing diagnosis of HTN. In patients without baseline HTN, the rates of incident HTN were 23.4, 26.6, and 28 (*p* > 0.2). It was described that in all participants, the random assignment to canakinumab did not reduce the BP (*p* > 0.2) or incident HTN during the follow-up period (hazard ratio, 0.96 [0.85–1.08], *p* > 0.2). However, it did reduce the rates of major adverse cardiovascular events. Therefore, these analyses suggest that the mechanisms underlying this benefit are not related to changes in BP or incident HTN [149].

### 4.3. The Role of the Gut Microbiome in Immune Regulation and Hypertension Therapy

Modulating the gut microbiome is gaining recognition as a promising strategy for managing hypertension. Evidence from various sources, including both animal models and initial human studies, increasingly points to the effectiveness of specific interventions. These interventions, which include the intake of dietary fiber and probiotics, have shown potential in contributing to lower blood pressure levels. This area of research suggests that by influencing the composition and function of the gut microbiota, we might open new avenues for therapeutic approaches to hypertension [150].

#### 4.3.1. Fiber

Dietary fiber, defined as undigestible carbohydrate polymers with a degree of polymerization [151], has been studied in recent years due to its effect on blood pressure, mediated by its relationship with the gut microbiome. The microbiome ferments certain types of fiber, releasing microbial metabolites called short-chain fatty acids (SCFAs), such as acetate, propionate, and butyrate [152]. These can also trigger various downstream signaling pathways, either by activating G protein-coupled receptors or by directly stimulating immune cells [153].

Clinical studies have been conducted to evaluate the effects of SCFAs. Among them is a Phase II, randomized, placebo-controlled, and double-blind crossover trial that examined the impact of a prebiotic supplement: acetylated and butyrylated high-amylose maize starch (HAMSAB). In this study, 20 participants with untreated hypertension received either 40 g/day of HAMSAB or a placebo for three weeks, with a three-week washout period between treatments. The results revealed that participants who received HAMSAB experienced a clinically significant reduction in their 24 h systolic blood pressure, but no alterations were observed in the plasma levels of cytokines, including IL-1β, IL-17A, IL-10, and TNF-α [154].

Another study, a blinded, randomized crossover dietary intervention, aimed to determine whether increasing the dietary intake of SCFAs could influence adaptive immunity in humans. It analyzed twenty healthy adults, who consumed a high-SCFA diet for 21 days. The high-SCFA diet significantly boosted fecal SCFA concentrations while reducing fecal ammonia. It also led to notably higher plasma levels of propionate and butyrate. Concurrently, this diet resulted in significantly lower counts of several circulating immune cells, including total B cells, naive B cells, Th1 cells, and mucosal-associated invariant T cells [155].

However, the critical evaluation of this field reveals two major controversies: the causality and clinical relevance of the observed effects. While a fecal microbiota transplantation in animal models supports a causal role, human data remain largely associative. A fundamental challenge lies in determining whether dysbiosis causes or results from hypertension. The modest blood pressure reductions reported in meta-analyses (typically 2–4 mmHg), though promising, must be contextualized against established pharmacotherapies. Notably, Reynolds et al.’s comprehensive meta-analysis of 185 observational studies and 58 clinical trials (∼135 million person-years) found that fiber consumption was associated with a 15–30% lower cardiovascular mortality risk and a 1.27 mmHg SBP reduction [156], highlighting potential population-level benefits despite individual effect sizes.

A key research gap remain—the lack of large-scale, long-term clinical trials demonstrating whether microbiome modulations through fiber or probiotics can produce clinically relevant and sustained blood pressure reductions. The current evidence, while mechanistically intriguing, has yet to establish microbiome-targeted interventions as definitive hypertension therapies, warranting both cautious interpretation and further investigation.

#### 4.3.2. Probiotics

Recent findings suggest that an increased probiotic intake is linked to a reduced risk of chronic metabolic disorders, like hyperglycemia, dyslipidemia, and hypertension. This could prove promising as a therapeutic alternative to currently available pharmaceutical treatments [157].

An umbrella meta-analysis led by Zarezadeh et al., encompassing 14 meta-analyses and over 15,000 participants, found that probiotic supplementation significantly reduced both SBP and DBP. Reductions were more pronounced for SBP in older individuals (over 50 years old) and with shorter intervention durations (≤10 weeks) and for DBP with higher probiotic dosages (≥10^10^ CFU). Notably, SBP also decreased more in patients with pre-existing hypertension or diabetes [158].

Similarly, Khaleesi et al. conducted a systematic review and meta-analysis of nine randomized, controlled trials to explore the impact of probiotic consumption on blood pressure, revealing a significant average reduction of 3.56 mmHg in SBP and 2.38 mmHg in DBP. These modest improvements were more pronounced with multiple probiotic species, in individuals with an elevated baseline BP (≥130/85 mmHg), and with interventions lasting at least 8 weeks at daily doses of ≥10^11^ colony-forming units (CFUs), suggesting that under specific conditions, regular probiotic intake could be a beneficial strategy for BP management [159].

Likewise, a meta-analysis and systematic review conducted by Chi et al. highlights a significant inverse association between a probiotic intake and the prevalence of chronic metabolic disorders, such as hypertension, dyslipidemia, and elevated blood glucose. They analyzed 14 studies involving 846 participants; a reduction was observed in the SBP (2.05 mmHg) and DBP (1.26 mmHg). Beneficial effects were also found in reducing the BMI (1.03) and blood glucose levels (0.18 mmol/L). The positive effects on BP were influenced by the treatment duration, the dosage, and the participant’s age, with particular benefits seen in individuals with diabetes mellitus [160].

### 4.4. Vaccination as an Innovative Therapeutic Strategy for Hypertension

On the other hand, an innovative proposal is the development of a vaccine against HTN, with several models currently under study, all targeting RAS components. For example, the Ang I-R vaccine against the endogenous peptide Ang I has shown good results in mice, reducing BP levels as well as vascular and kidney damage. While still in preclinical stages, such approaches could revolutionize long-term hypertension management by offering a sustained immunological modulation of key pathways like the RAS, potentially reducing the need for daily pharmacotherapy. Further research is needed to validate the safety and efficacy in humans [161].

Therefore, building on these promising initial findings related to the immunological targeting of hypertension, a key priority for future research is to elucidate the precise mechanisms by which these novel therapeutic strategies exert their blood pressure-lowering effects. While initial evidence strongly suggests their benefit, a deeper understanding of the specific cellular and molecular pathways involved is currently lacking. Unraveling these underlying mechanisms will allow us to optimize our current therapeutic alternatives.

## 5. Conclusions

HTN is a major public health problem. It is a significant risk factor for the development of coronary, cerebrovascular, and peripheral diseases. Recent data have shown a strong association between HTN and the inflammatory response, where various stimuli—including Ang II and sodium, to name a few—are capable of inducing a response from the immune system (SI) by initiating a signaling cascade that leads to the expression of pro-inflammatory genes with the subsequent production of pro-inflammatory cytokines (TNF-α, IL-1β, IL-6, IFN-g, and IL-17A) and the activation of adaptive immunity. This process also increases immune cells’ infiltration into blood vessels, perivascular adipose tissue, kidneys, and the nervous system, leading to a process of remodeling and dysfunction, and as they become dysfunctional, their roles in regulating BP are affected. Based on this, the immune system is proposed as a potential therapeutic target for HTN, highlighting drugs such as MMF and MTX or even monoclonal antibody therapy. However, currently, there are no clinical trials that have approved the efficacy, safety, and adverse effects of these in humans. For this reason, it is suggested that these aspects be explored further to propose therapeutic alternatives for this disease.

## Figures and Tables

**Table 1:** Hypertension as an immunological disease: evidence over time.

Authors	Methodology	Results
Ebringer et al. [14]	A case–control study measuring IgG levels in 118 patients with severe hypertension and 163 normotensive individuals.	Serum IgG levels were significantly higher in 118 patients with severe hypertension compared to a group of 163 normotensive blood donors.
Adlin et al. [15]	Case–control study measuring serum immunoglobulin levels in 52 hypertensive patients and 52 normotensive controls.	Contrary to previous reports, hypertensive subjects did not have higher levels of IgG or IgA than controls. The authors attributed this to the mild elevation of blood pressure.
Mirhafez et al. [16]	A case–control study measuring blood levels of 12 cytokines and growth factors in 155 individuals with hypertension and 148 normotensive individuals.	Hypertensive subjects had higher concentrations of IL-1α, -2, -8, TNF-α, IFN-γ, MCP-1, EGF, and VEGF. They also had lower levels of the anti-inflammatory cytokine IL-10 (*p* < 0.05) compared to healthy individuals.
Sesso et al. [17]	Prospective cohort study starting in 1992 with 20,525 U.S. healthcare professionals aged 45 or older, aiming to examine CRP levels.	During follow-up, 5365 women developed hypertension. C-reactive protein was significantly associated with an increased risk of developing hypertension in all prespecified subgroups evaluated, including those with very low baseline blood pressure and those without traditional coronary risk factors.
Bautista et al. [18]	A cross-sectional study in 300 individuals evaluating whether circulating CRP levels are independently related to essential hypertension.	Plasma CRP level is an independent risk factor for hypertension. The unadjusted prevalence of hypertension was 58.7% in the highest quartile of CRP, compared to only 34.7% in the lowest quartile.
Youn et al. [19]	Case–control study evaluating renal cell infiltration through immunohistochemical staining in kidney biopsy samples from 71 patients with hypertensive nephrosclerosis and 71 control subjects.	Higher numbers of CD4+ and CD8+ T cells were found infiltrating the tubulointerstitial system of hypertensive nephrosclerosis patients compared to normotensive control subjects.
Navarro et al. [20]	Case–control study evaluating the relationship between inflammatory parameters (CRP, serum, and urinary TNF-α) and markers of preclinical TOD (LVH and microalbuminuria) in 40 newly diagnosed, never-treated essential hypertension patients, compared to 21 healthy controls.	Urinary TNF-alpha is independently correlated with urinary albumin excretion, suggesting inflammation may contribute to TOD development. Additionally, urinary TNF-α excretion could be an early marker of preclinical TOD in hypertensive patients.

Abbreviations: IL (interleukin); TNF-α (tumor necrosis factor-alpha); IFN-γ (interferon-gamma); MCP-1 (monocyte chemoattractant protein-1); VEGF (vascular endothelial growth factor); EGF (epidermal growth factor); CRP (C-reactive protein); LVH (left ventricular hypertrophy); MAB (microalbuminuria); and TOD (target organ damage).

## Data Availability

No new data were created or analyzed in this study. Data sharing is not applicable to this article.
